# Immunoglobulin G4-Related Disease of the Intestine: A Clinicopathological Entity to Be Considered

**DOI:** 10.3390/medicina60010057

**Published:** 2023-12-28

**Authors:** Filippo Vernia, Laura Cirella, Giuseppe Calvisi, Angelo Viscido, Giovanni Latella

**Affiliations:** 1 Department of Life, Health, and Environmental Sciences, Division of Gastroenterology, Hepatology, and Nutrition, University of L’Aquila, Piazza S. Tommasi, 1, Coppito, 67100 L’Aquila, Italy; filippo.vernia1@gmail.com (F.V.); angelo.viscido@univaq.it (A.V.); 2 Pathology Unit, San Salvatore Hospital, Via Lorenzo Natali, 1, Coppito, 67100 L’Aquila, Italy; laura.cirella88@gmail.com (L.C.); gcalvisi@asl1abruzzo.it (G.C.)

**Keywords:** immunoglobulin G4-related disease, IgG4-RD, inflammatory bowel diseases, Crohn’s disease, intestinal strictures

## Abstract

*Background and Objectives*: Immunoglobulin G4-related disease (IgG4-RD) is a recently recognized immune-mediated, systemic condition of unknown etiology, associated with fibroinflammatory lesions. Diagnosis is set in the presence of IgG4-positive plasma cell infiltration of the involved tissue and elevated serum IgG4 levels. However, approximately 30% of patients have normal serum IgG4 levels. IgG4-RD may affect several organs, including the pancreas, bile ducts, mesentery, retroperitoneum, and salivary glands, but the involvement of the gastrointestinal tract is uncommon. *Materials and Methods*: The case series of 4 patients with IgG4-RD involving the intestinal tract was observed in the period of 2017–2022: One of these 4 cases has already been published previously as a case report. Colorectal and ileal biopsy specimens were stained with hematoxylin and eosin and immunohistochemical techniques using monoclonal antihuman IgG4 primary antibody. Diagnosis of IgG4-RD was based on the presence of >50 cells/ HPF and IgG4/IgG ratio >40 confirmed by two pathologists. *Results*: IgG4-RD was set in patients previously diagnosed as affected by Crohn’s disease. *Conclusions*: Systematic IgG4 immunohistochemical staining should be considered in the diagnostic workup of patients with gastrointestinal strictures, mimicking Crohn’s disease. The exact prevalence of the condition is likely more frequent than reported and should be defined by a large series of consecutive patients.

## 1. Introduction

Immunoglobulin G4-related disease (IgG4-RD) is a recently recognized immune-mediated, systemic, inflammatory condition of unknown etiology [1,2,3,4,5]. First described as autoimmune pancreatitis [6,7,8], it consists of a wide range of disorders that involve a variety of organs including the urinary tract [9], liver and bile ducts [7], skin [10], pleura, peritoneum, and meninges [2,4,5]. They may also occur in the absence of concomitant pancreatic disease [1,10,11]. The entire gastrointestinal tract may be affected, resulting in clinical and histological features that mimic Crohn’s Disease (CD) [1,11,12]. Stenosis is a common complication of several neoplastic and inflammatory diseases, like intestinal IgG4-RD and CD [1].

Shared characteristics of IgG4-RD include tumor-like swelling of the affected organs, lymphoplasmacytic infiltrate characterized by IgG4-positive plasma cells, variable degree of fibrosis, described as storiform fibrosis, artery-sparing obliterative phlebitis, perineuritis, and mild tissue eosinophilia [13,14,15,16]. High serum concentrations of IgG4 are present in about half of IgG4-RD patients [13,17].

Some of these features such as the predominance of lymphocytes and plasma cells versus acute inflammatory cells, and full-thickness fibrosis are frequent in CD, also in the absence of diagnostic histologic features such as non-epithelioid granulomas. Arterial-sparing perivenulitis and perineural chronic inflammation are also present in some patients [18].

Several recent papers reported the presence of IgG4-related intestinal stenosis that presented with clinical and endoscopic features similar to IBD, but despite the increasing evidence systematic IgG4 immunohistochemical staining is usually not considered in the diagnostic workup of patients with gastrointestinal strictures [19,20,21,22,23,24].

The aim of the present paper, reporting four cases of IgG4-RD first misdiagnosed as CD, is to highlight the importance of this recently recognized pathology in the diagnostic workup of non-neoplastic intestinal strictures.

## 2. Materials and Methods

The diagnosis of IgG4-related gastrointestinal disease was made in endoscopic biopsies or surgical specimens from the terminal ileum and large bowel using standard histological staining and immunohistochemical techniques in four patients observed in the period 2017–2022, first diagnosed as affected by Crohn’s disease (CD). One of these 4 cases has already been published previously as a case report [1], to which some new information has been added and included for completeness in this case series.

Colorectal and ileal specimens were treated with 4% formaldehyde solution in phosphate buffer saline, pH 7.4, at room temperature, and paraffin-embedded for histopathological evaluation. Two pathologists (LC, GC) independently reviewed 3 µm sections under a Zeiss Axio Imager A2 Light Microscope (Carl Zeiss Microscopy, LLC, One Zeiss Drive, Thornwood, NY, USA) after staining with standard hematoxylin and eosin (H&E). Sections were cut from the paraffin block, mounted on charged slides, and placed into a Dako OMNIS system (Dako Denmark A/S, Produktionsvej 42, Glostrup, Denmark). Immunohistochemical techniques using monoclonal antihuman IgG4 primary antibody at 1:200 dilution (clone HP6025–A10651, Invitrogen, Molecular Probes Inc., 29851 Willow Creek Road, Eugene, OR, USA) were used. IgG4-positive plasma cells were counted in 400 (40×) high power fields (HPFs) in the section with the highest cellular density. The presence of >50 cells/ HPF as well as ratio a IgG4/IgG > 40 was required for diagnosis of IgG4-RD.

## 3. Detailed Case Description

### 3.1. Case 1

A 38-year-old woman had for the first time in 2017 had severe, acute right lower abdominal pain, abdominal tenderness suggesting acute abdomen, fever, and severe leukocytosis. The patient went to the Emergency Department of another institution and laparoscopy was performed with a suspected diagnosis of acute appendicitis. During the procedure the appendix was normal, but a 10 cm caecal mass was detected with marked wall thickening of the terminal ileum, mimicking neoplasia. The patient underwent a right hemicolectomy. CD was diagnosed on the surgical specimen, on the base of the site of the lesion, and suggestive histological findings consisting of the presence of transmural acute and chronic inflammatory infiltrate, associated with fibrosis. The patient was referred to our IBD center, but being asymptomatic, never came to visit. Three months following surgery she reported mild abdominal pain and non-bloody diarrhea, in the absence of fever, arthralgia, or other symptoms. During the first consultation, the abdomen was slightly tender, with no other abnormal findings. Erythrocyte sedimentation rate (ESR), and C-reactive protein (CRP) were 34 mm/h and 0.17 mg/dL, respectively.

Ileo-colonoscopy and upper endoscopy with multiple biopsies were performed, to confirm or challenge the diagnosis of CD, and investigate the possible involvement of other intestinal segments. The endoscopic findings were normal, the ileo-colonic anastomosis included. The histological examination of the biopsy specimens could not support the diagnosis of CD but showed diffuse perivenulitis and perineuritis which prompted immunohistochemical IgG4 staining.

Histological specimens of pre-anastomotic ileal-, colonic-, and rectal mucosa showed some IgG4+ plasma cells in the capillaries, as well as in the lamina propria. Biopsies of the upper GI tract showed normal duodenal mucosa with slightly increased IgG4+ plasma cells. Several IgG4 deposits were conversely found in the lumen of capillaries. There was no evidence of IgG4+ plasma cells in gastric mucosa. The esophagus was histologically normal.

The intestinal specimens resected during surgery were re-evaluated blindly by two experienced pathologists (L.C. and G.C.) as follows.

**Figure 1:**
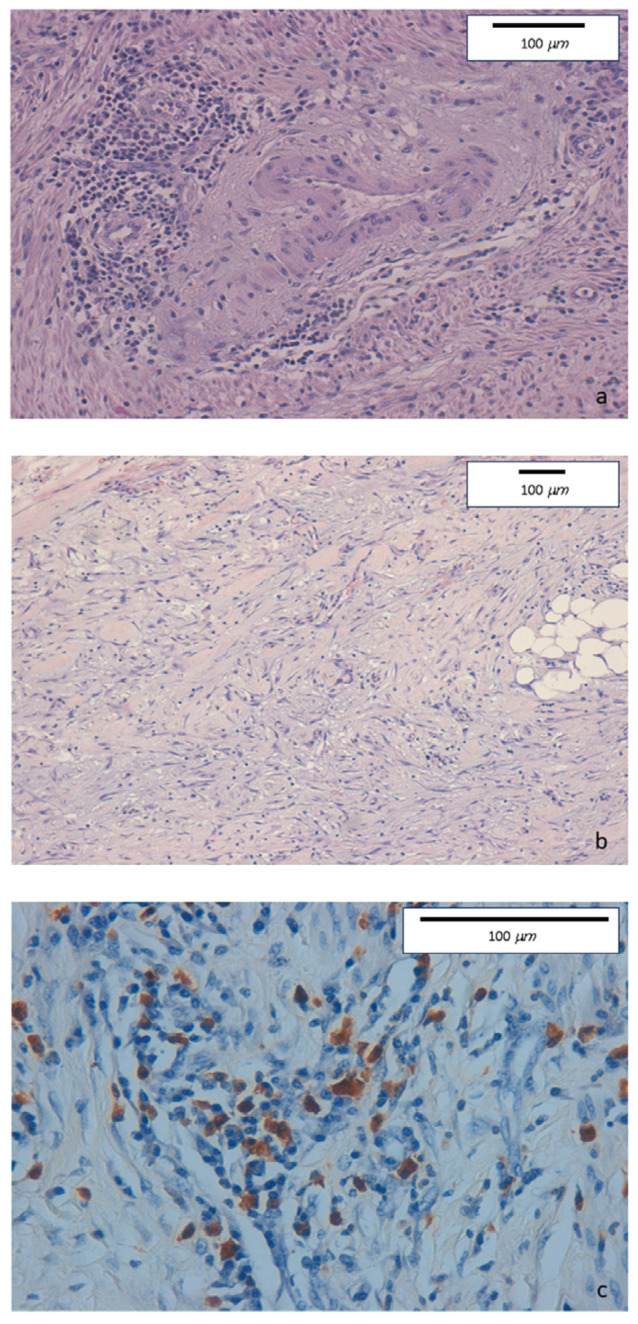
(**a**) Haematoxylin-eosin staining of colonic specimens. Original magnification 20×. Dense inflammatory lymphoplasmacytic infiltrate involving venous structures but sparing arterial vessels. (**b**) Haematoxylin-eosin staining of colonic specimens. Original magnification 10×. Diffuse sclerosing fibrosis with storiform aspect and lymphoplasmacytic infiltration. (**c**) Immunohistochemical staining of colonic specimens. Original magnification 40×. Colonic mucosa showing dense inflammatory lymphoplasmacytic infiltration, with a high prevalence of IgG4-positive plasma cells (≥50/HPF).

The intense inflammatory lymphoplasmacytic infiltrate was present in the ileocecal valve and in the right colon biopsies. Some eosinophils, neutrophils, and nodular lymphocytic aggregates in the absence of epithelioid granulomas were observed in the epithelial layer. The inflammatory infiltrate was particularly dense in the submucosa, muscular layer, and serosa. Marked fibrosis and edema were detected in submucosa, serosa, and pericolic tissue. Fibrin and granulocytes were found in the serosa. Perivenulitis with arteriolar sparing, and perineuritis with nervous fiber damage were prominent. A significantly increased number of IgG4-type plasma cells was documented by immunohistochemical staining (80 per HPF). The IgG4+/IgG ratio was 50% (Figure 1). The new re-evaluation of this patient’s surgical specimen according to the current histopathological criteria confirmed the previous diagnosis of IgG4-related intestinal disease.

A total-body CT scan did not show any other lesion. Bowel ultrasonography was negative.

IgG4 serum levels were within the normal range (46 mg/dL). Autoimmune disease or vasculitis were excluded (anti-nuclear antibodies, anti-mitochondrial antibodies, anti-smooth muscle antibodies, anti-liver kidney microsomal antibodies, perinuclear anti-neutrophil cytoplasmic antibodies, anti-Saccharomyces cerevisiae antibodies, and anti-cyclic citrullinated peptide), as well as celiac disease (anti-tissue transglutaminase antibodies and anti-endomysial antibodies) or an infection (Mycobacterium tuberculosis, Epstein–Barr virus, Cytomegalovirus). No involvement of the pancreas or biliary tract was present.

The clinical and histopathological picture suggested the diagnosis of IgG4-related disease with involvement of the distal ileum and right colon.

In view of the few clinical symptoms and the absence of significant intestinal lesions, no drug treatment was suggested. Six years after surgery, the patient reported feeling well with no significant intestinal symptoms. The absence of significant new intestinal lesions and the persistence of clinical remission for such a prolonged period suggests a more favorable and less aggressive clinical course of IgG4-related disease of the intestine compared to that of Crohn’s disease.

### 3.2. Case 2

A 34-year-old man was first diagnosed in 2005 as affected by CD of the ileum, caecum, and sigmoid colon in a different institution (Crohn’s disease endoscopic index of severity 11). The onset of the disease was characterized by diarrhea (4–5 bowel movements of loose stool) and abdominal pain. CRP was slightly elevated at 14.8 mg/dL, and the patient underwent a small bowel ultrasound, showing 15 cm-long mild ileal thickening. Symptoms subsided following short-term steroid treatment, and therapy was discontinued.

The following year the same symptoms recurred, progressively worsening over a few weeks. The patient underwent a CT scan. An ileal sub-stenosis, inflamed appendix, and cecal abscess were diagnosed. An ileocecal (20 + 30 cm) resection was thus performed. The resected specimen was suggestive of CD. After surgery, the patient reported long-lasting, complete wellbeing.

In January 2018 the patient was visited once more in the emergency department, due to persistent abdominal pain and profuse acute diarrhea. CRP was high, 42.8 mg/dl as well as fecal calprotectin (204 mg/kg). A colonoscopy was performed, showing inflammation of the anastomosis and sub-stenosis of the neo-terminal ileum (Rutgeert’s i4). Histological evaluation of terminal ileum and large bowel biopsies were suggestive of CD with high-grade granulocyte infiltrate. A 20 cm long sub-stenosis was confirmed by MRI.

In June 2018, the patient was referred to our IBD center. The symptoms consisted of persistent mild non-bloody diarrhea (3 daily bowel movements) and mild abdominal pain. Blood cells count was within the normal range and CRP 12.3 mg/dL.

Tissue specimens of the previous surgical specimen was re-evaluated by a GI-dedicated pathologist, as well as the biopsies taken during the colonoscopy.

**Figure 2:**
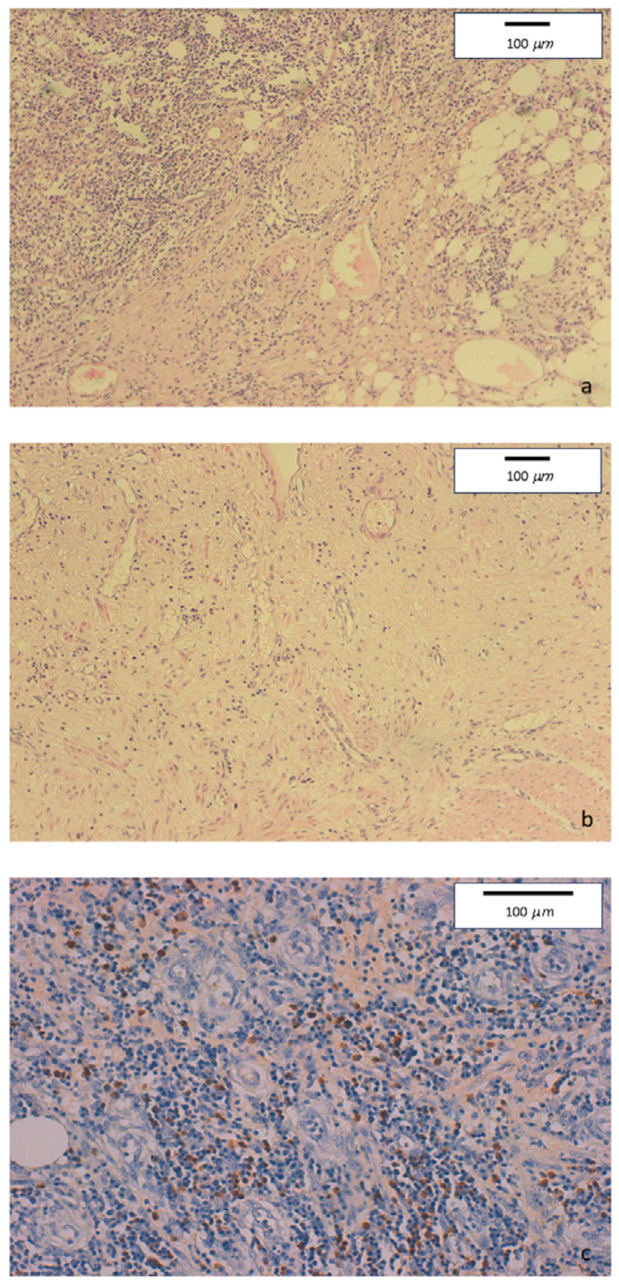
(**a**) Hematoxylin-eosin staining of colonic specimens. Original magnification 10×. Diffuse inflammatory lymphoplasmacytic infiltrate with perineural involvement. (**b**) Hematoxylin-eosin staining of colonic specimens. Original magnification 10×. Severe fibrosis with low tissue cellularity and lymphoplasmacytic infiltration, associated to edema of the lamina propria. (**c**) Immunohistochemical staining of colonic specimens. Original magnification 20×. Colonic mucosa showing dense inflammatory lymphoplasmacytic infiltration involving venous structures, with marked increase of IgG4-positive plasmacells (≥50/HPF).

Mucosal erosions, ulcers, and diffuse lymphoplasmacytic infiltrate were present in the ileo-colonic surgical specimen. Few full thickness, large nodular aggregates were also observed. Submucosa, serosa, and pericolic adipose tissue showed edema and fibrosis. Some foreign body, giant cell granulomas were present within submucosal nodular aggregates, but no epithelioid granuloma. Diffuse perivenulitis with arteriolar sparing was present, as well as perineuritis primarily involving the plexus od Auerbach. Immunohistochemistry showed a large number of IgG4+ plasma cells (70 per HPF) in some HPF with 40% IgG4/IgG rate (Figure 2).

Ileal biopsies, obtained during colonoscopy, were characterized by lymphoplasmacytic infiltrate, with few eosinophils. Minimal villous distortion was also present, with normal villus/crypt ratio. The muscularis mucosae was normal. Several IgG4+ plasma cells were present in the lamina propria, as well as IgG4 deposits within the capillaries.

A diagnosis of IgG4-related disease was set.

The upper endoscopy with biopsies showed mild chronic, non-specific HP-negative gastritis and normal duodenal mucosa.

A complete panel of autoimmunity tests was normal.

The patient was treated with budesonide 9 mg/day for 6 weeks leading to complete remission of symptoms.

### 3.3. Case 3

An 18-year-old woman first reported bloody diarrhea in 2007. High ESR (72 mm/h) and CRP (99 mg/dL) were also present. The blood cell count was within the normal range. The stool examination for parasites and bacteria was negative, and the occult blood test was positive.

A colonoscopy showed severe inflammation of the ileo-cecal valve, which prevented the examination of the terminal ileum. Ulcers were observed in the transverse, descendent, and sigmoid colon, with edema and hyperemia of the surrounding mucosa. The rectum was spared. Histological examination of the biopsies suggested active CD. A therapy with oral mesalamine 3.2 g/day and prednisone 50 mg/day was prescribed, and symptoms rapidly subsided.

In the following six years, three more clinical relapses were effectively treated with prednisone. In 2014, the intestinal MRI documented a 15 cm-long thickening (11 mm) of the terminal ileum.

In 2014, due to a relapse consisting of bloody diarrhea and abdominal pain, a colonoscopy was performed. The examination was limited to the rectum, sigmoid, and part of the descending colon due to stenosis, which was not previously present (simple endoscopic score for Crohn’s disease 12). Biopsies of the stenosis were consistent with the diagnosis of CD. Following effective steroid treatment, complete clinical remission was maintained with sulfasalazine 2 g/day and a 4-year course of azathioprine 100 mg/day.

In March 2018, despite the ongoing therapy, diarrhea and abdominal pain reappeared and the patient was referred to our IBD tertiary center. A colonoscopy confirmed stricture of the distal descending colon, associated with ulcerations and mild inflammation of the sigmoid and rectal mucosa. CT scan showed a 4 cm-long slight parietal thickening (13 mm) in the descending colon.

**Figure 3:**
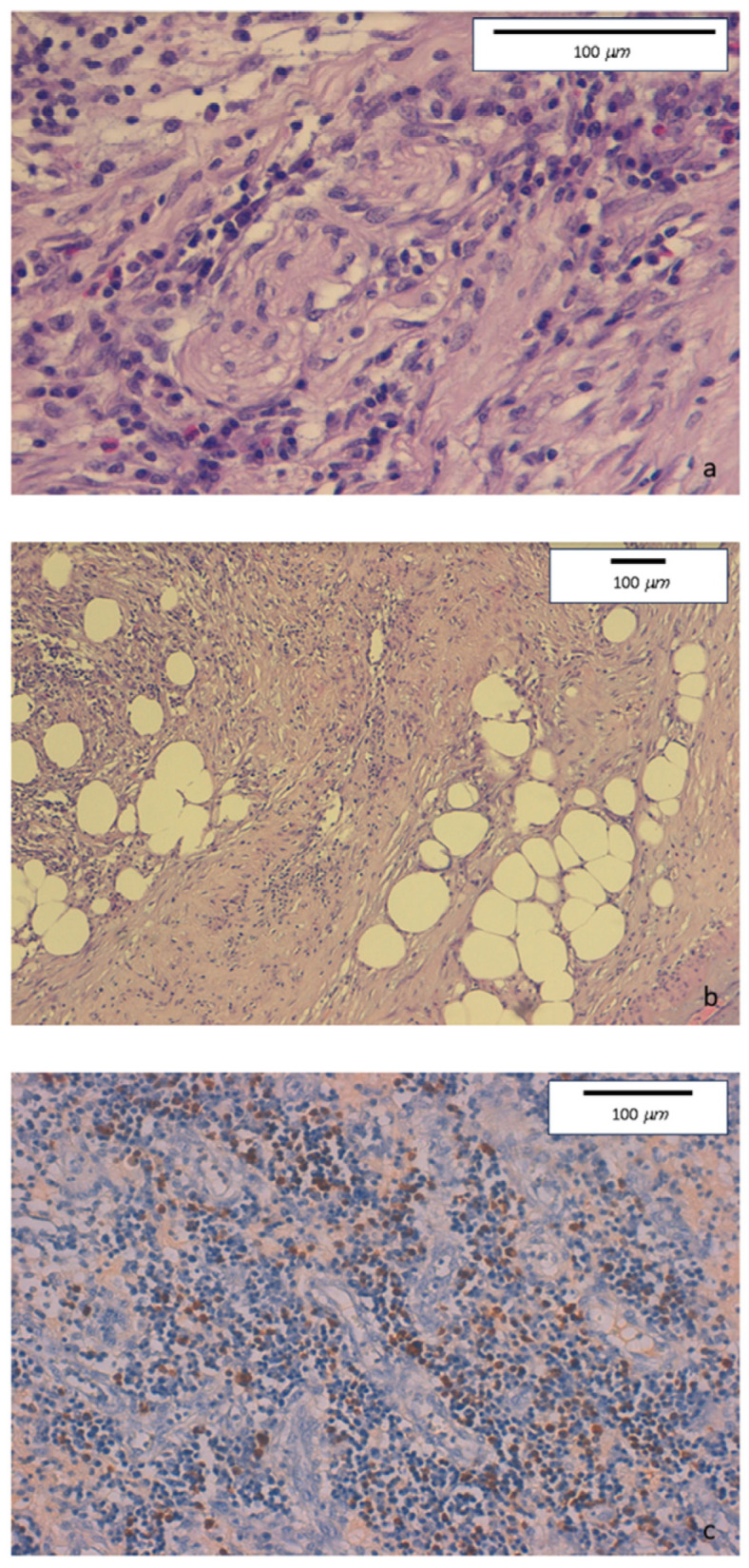
(**a**) Hematoxylin-eosin staining. Original magnification 40×. Intense lymphoplasmacytic inflammatory infiltrate with perineuritis and fibrous bands. (**b**) Hematoxylin-eosin staining. Original magnification 10×. Inflammatory infiltrate involving venous structures, in the presence of severe fibrosis with some storiform features. (**c**) Immunohistochemical staining. Original magnification 20×. Colonic mucosa showing diffurse perivenular lymphoplasmacytic inflammatory infiltration, with numerous IgG4-positive plasma cells (≥50/HPF).

The histologic evaluation of descending colon biopsies showed acute erosive colitis with marked lymphoplasmacytic infiltration associated with neutrophil and rare eosinophil granulocytes. No granulomas were found. Conversely, perineuritis and perivenulitis were detected, with arteriolar sparing. Marked IgG4+ plasma cell infiltration was detected in the colonic mucosa (Figure 3).

Based on these histological findings the diagnosis of CD was revised and instead established that it was an IgG4-related disease involving the colon.

A short course of steroids led to an improvement in diarrhea. The persistence of abdominal pain endoscopic pneumatic dilatation of the short left colonic stricture or surgery is currently under evaluation.

### 3.4. Case 4

An eighty-year-old woman presented to our Emergency Department in June 2022 with intermittent acute abdominal pain and vomiting suggestive of intestinal sub-occlusion. An abdominal CT scan showed thickening of the ileal and cecal walls. Non-homogeneous enhancement and lymph nodes increased in size up to 15 mm were also described. Colonoscopy showed intense inflammation of the caecum and one deep ulceration, near the stenotic ileo-cecal valve (simple endoscopic score for Crohn’s disease 7). Colonic biopsies showed acute and chronic non-specific inflammation. Blood tests showed only moderate increases in leukocytes, ESR, and CRP. The parasitological and bacteriological examination of the stools was negative. An antibiotic treatment with ciprofloxacin 1 g/day and metronidazole 1 g/day was started. After 3 days, as the abdominal pain and vomiting persisted, the laparoscopic surgical resection of approximately 20 cm of the distal ileum and cecum was performed.

**Figure 4:**
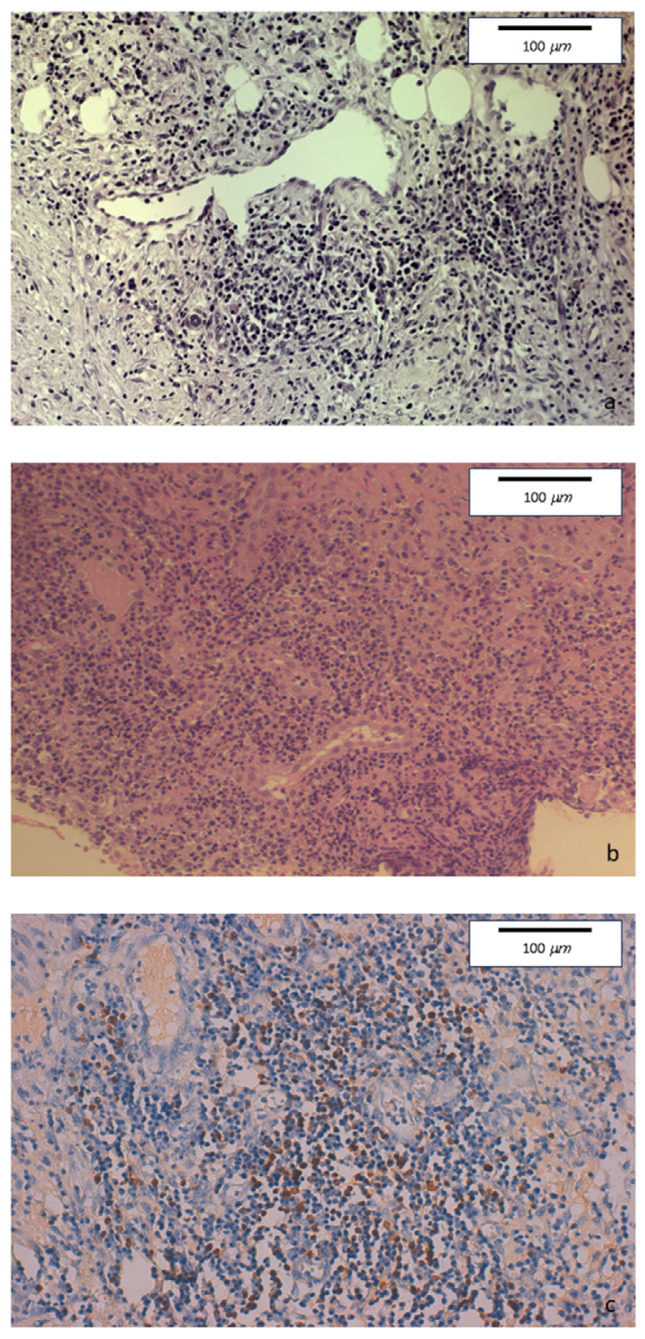
(**a**) Hematoxylin-eosin staining of colonic specimens. Original magnification 20×. Dense inflammatory lymphoplasmacytic infiltration, with initial storiform fibrosis. (**b**) Hematoxylin-eosin staining. Original magnification 20×. Diffuse lymphoplasmacytic inflammatory infiltrate also involving venous and lymphatic structures. (**c**) Immunohistochemical staining. Original magnification 20×. Colonic mucosa showing perivenous and perilymphatic dense inflammatory lymphoplasmacytic infiltration, with a high number of IgG4-positive plasma cells (≥50/HPF).

The histopathologic examination of the surgical specimen showed chronic sclerosing ileo-colitis, involving mucosa and submucosa, mainly consisting of lymphoplasmacytic with few eosinophils. Perivenulitis without arterial damage and perineuritis were also present. Marked fibrosis was identified. An increase in IgG4+ plasma cells was detected as well as the deposition of IgG4 in the blood vessels and in sclerohyaline areas (Figure 4).

Based on these histological findings, the diagnosis of IgG4-related ileocecal disease was made. After the surgery, the patient recovered quickly without having any intestinal symptoms. No prophylactic medical treatment was prescribed.

Six months after surgery, the patient then underwent a follow-up colonoscopy which found no macroscopic lesions of the mucosa of the distal ileum, colon, and rectum nor alterations in the caliber of the intestinal lumen. Histological examination of the biopsies revealed the persistence of slightly increased IgG4+ plasma cells in the distal ileum and in the colon. An upper endoscopy with biopsies revealed only a mild non-specific inflammation of the gastric antrum.

## 4. Discussion

IgG4-related disease is an immune-mediated, systemic, inflammatory condition often associated with autoimmune pancreatitis (AIP) and IgG4-related sclerosing cholangitis. According to the most recent guidelines [14,25,26] IgG4-RD is diagnosed when there is a localized or diffuse swelling or mass involving a single or multiple organs, associated with elevated serum IgG4 levels, local infiltration of IgG4+ plasma cells and lymphocytes, as well as storiform fibrosis and obliterative phlebitis [14,15,16]. However, approximately 30% of patients have normal serum IgG4 levels [13,17,27]. An increased number of IgG4+ plasma cells has also been described in ulcerative colitis (UC) both with and without concomitant sclerosing cholangitis (26.6% and 5%, respectively) [28,29]. Similarly, increased numbers of IgG4+ plasma cells were reported also in biopsies of Crohn’s disease patients, and a cut-off > 15 IgG4+ cells was associated with increased fibrosis (*p* = 0.039) [30].

The cut-off values used in different studies range from >10 to >50 IgG4+ cells per HPF. The most widely accepted diagnostic criteria for IgG4-RD consists in the presence of >50 IgG4+ plasma cells per HPF associated with IgG4+/IgG ratio > 40% [2,14,15,16]. Colonic or ileo-colonic involvement in the absence of AIP or IBD is rare [1,31,32,33] and difficult to diagnose unless actively sought. Moreover, mimicking malignancy and stenosing CD, intestinal IgG4-RD in most instances is diagnosed following surgery [1,32]. Being rare and requiring IgG4 immunohistochemical staining that is not routinely performed, only a few cases were successfully diagnosed preoperatively [22].

Measurement of the serum IgG4 levels could be helpful, but, again, the measurement is rarely performed. Moreover, a large proportion of patients affected by documented IgG4-RD has normal levels of circulating IgG41 and only a high level of suspicion prompts IgG4 immunohistochemical staining of endoscopic biopsies and surgical specimens. In the presence of an intense lymphoplasmacytic infiltrate, storiform fibrosis, an artery-sparing obliterative phlebitis, and a perineuritis with nervous fiber damage the pathologist should always consider the presence of a possible IgG4-related disease. These features might be misdiagnosed by non-GI-dedicated pathologists for Crohn’s disease, as lymphoplasmacytic infiltrate and fibrosis occur also in this condition. The presence of storiform fibrosis, arterial sparing obliterative phlebitis, and perineuritis represent the main discriminant between the two pathologies, prompting immunostaining.

Early and correct diagnosis of IgG4-related disease and early steroid therapy could limit unnecessary surgery. However, some patients with IgG4-RD develop strictures despite medical treatment [1]. Management of mild IgG4-RD is different from that of Crohn’s disease, usually consisting of short courses of steroids. Prevention of relapse is achieved with both steroids or traditional immunomodulators, while only a few cases need B-cell depletion therapy with anti-CD20 antibodies. The efficacy of biologics and small molecules is instead less documented and might not be effective [14,16,34].

IgG4-related disease involving the intestine often mimicking appendiceal tumor or appendicitis, Crohn’s Disease, sclerosing mesenteritis, intestinal neoplasia, malignant bowel obstruction, and multifocal ulcerating stenosing enteritis [35,36,37,38].

IgG4-related disease of the intestine, in many cases, was diagnosed postoperatively for presumed appendicitis, CD, or tumor. Most patients were treated with surgical or endoscopic resection, whereas a minority received glucocorticoids and/or immunosuppressants. Some patients exhibited good treatment response to glucocorticoid therapy, though it was incomplete in long-standing disease, which could be explained by the prominent fibrotic component of the injury. In a few cases, patients received maintenance treatment with immunosuppressive agents (mycophenolate, cyclosporine, and azathioprine), which appeared effective [1,14,16,20,39,40,41]. Careful monitoring of patients is required, as in the clinical course of IgG4-related disease, further lesions may appear as late as years after initial manifestation is diagnosed and could be located in different organs [14,16].

## 5. Conclusions

Gastrointestinal IgG4-RD often mimics CD or intestinal neoplasia, and diagnosis is difficult. Systematic IgG4 immunohistochemical staining should routinely be performed on the gastrointestinal biopsies of patients first presenting with Crohn’s-like strictures to provide hard data on the real prevalence of this condition, which cannot be estimated by the present case series. The same applies to the surgical specimens of patients operated upon for gastrointestinal stenosis or mass, whenever neoplasia is not confirmed.

The update of our case series suggests that IgG4-related disease should be included in the diagnostic workup of patients with gastrointestinal strictures or mass, as its prevalence is likely underestimated, more so in patients with arterial sparing perivenulitis and perineuritis.

## Data Availability

Data are contained within the article.
