# Correction: Vernia et al. Immunoglobulin G4-Related Disease of the Intestine: A Clinicopathological Entity to Be Considered. *Medicina* 2024, *60*, 57

**DOI:** 10.3390/medicina62030486

**Published:** 2026-03-05

**Authors:** Filippo Vernia, Laura Cirella, Giuseppe Calvisi, Angelo Viscido, Giovanni Latella

**Affiliations:** 1 Department of Life, Health, and Environmental Sciences, Division of Gastroenterology, Hepatology, and Nutrition, University of L’Aquila, Piazza S. Tommasi, 1, Coppito, 67100 L’Aquila, Italy; filippo.vernia1@gmail.com (F.V.); angelo.viscido@univaq.it (A.V.); 2 Pathology Unit, San Salvatore Hospital, Via Lorenzo Natali, 1, Coppito, 67100 L’Aquila, Italy; laura.cirella88@gmail.com (L.C.); gcalvisi@asl1abruzzo.it (G.C.)

## Text Correction

The authors would like to correct some parts of the original manuscript [1] to avoid any misunderstanding of case 1 that was published in 2018 and properly quoted in the original publication as reference number 1 [2]. The corrected content appears below.

Abstract

The first sentence of the Materials and Methods is replaced as follows: “The case series of 4 patients with IgG4-RD involving the intestinal tract was observed in the period of 2017–2022: One of these 4 cases has already been published previously as a case report.”.

**2. Materials and Methods**

The first paragraph is replaced as follows: “The diagnosis of IgG4-related gastrointestinal disease was made in endoscopic biopsies or surgical specimens from the terminal ileum and large bowel using standard histological staining and immunohistochemical techniques in four patients observed in the period 2017–2022, first diagnosed as affected by Crohn’s disease (CD). One of these 4 cases has already been published previously as a case report [1], to which some new information has been added and included for completeness in this case series.”.

*3.1. Case 1*

The sentence “The intestinal specimen resected during surgery was re-evaluated as follows.” is replaced with this new one: “The intestinal specimens resected during surgery were re-evaluated blindly by two experienced pathologists (L.C. and G.C.) as follows.”.

At the end of the sentence “The IgG4+/IgG ratio was 50% (Figure 1).”, add the following new sentence: “The new re-evaluation of this patient’s surgical specimen according to the current histopathological criteria confirmed the previous diagnosis of IgG4-related intestinal disease”.

At the end of the sentence “In view of the few clinical symptoms and the absence of significant intestinal lesions, no drug treatment was suggested.”, add the following new sentence: “Six years after surgery, the patient reported feeling well with no significant intestinal symptoms. The absence of significant new intestinal lesions and the persistence of clinical remission for such a prolonged period suggests a more favorable and less aggressive clinical course of IgG4-related disease of the intestine compared to that of Crohn’s disease.”.

**5. Conclusions**

The last sentence “IgG4-related disease should be included in the diagnostic workup of patients with gastrointestinal strictures or mass, as its prevalence is likely underestimated, more so in patients with arterial sparing perivenulitis and perineuritis.” is replaced with this new sentence “The update of our case series suggests that IgG4-related disease should be included in the diagnostic workup of patients with gastrointestinal strictures or mass, as its prevalence is likely underestimated, more so in patients with arterial sparing perivenulitis and perineuritis.”.

The authors state that the scientific conclusions are unaffected. This correction was approved by the Academic Editor. The original publication has also been updated.
